# Idiopathic Environmental Intolerance Attributed to Electromagnetic Fields: A Content Analysis of British Newspaper Reports

**DOI:** 10.1371/journal.pone.0065713

**Published:** 2013-06-14

**Authors:** Buffy Eldridge-Thomas, G James Rubin

**Affiliations:** King’s College London, Department of Psychological Medicine, London, United Kingdom; National Research Council, Italy

## Abstract

Idiopathic environmental intolerance attributed to electromagnetic fields (IEI-EMF) is a controversial condition in which people describe symptoms following exposure to electromagnetic fields from everyday electrical devices. However, double-blind experiments have found no convincing evidence that electromagnetic fields cause these symptoms. In this study, we assessed whether recent newspaper reporting in the UK reflected this scientific evidence. We searched a database of newspaper articles to identify all those that contained IEI-EMF related keywords and selected a random sample of 60 for content analysis. For our primary outcomes, we assessed how many articles mainly or wholly presented an electromagnetic cause for IEI-EMF and how many discussed unproven treatments for the condition such as strategies intended to reduce exposure to electromagnetic fields or the use of complementary and alternative therapies. We also assessed whether the type of information source used by a newspaper article (e.g. scientist, person with IEI-EMF, politician) or the type of newspaper (broadsheet, tabloid, local or regional) was associated with either outcome. Of the 60 articles, 43 (71.7%) presented a mainly electromagnetic cause, compared to 13 (21.7%) which presented mainly non-electromagnetic causes and 4 (6.7%) which did not discuss a cause. 29 (48.3%) did not mention any potential treatment, while 24 (40.0%) mentioned eletromagnetic field related strategies and 12 (20.0%) mentioned complementary or alternative therapies. Articles which quoted someone with IEI-EMF were significantly more likely to report an electromagnetic cause and to present unproven treatments. Those which used a scientist as a source were more likely to present a non-electromagnetic cause for the condition. The widespread poor reporting we identified is disappointing and has the potential for to encourage more people to misattribute their symptoms to electromagnetic fields. Scientists should remain engaged with the media to counteract this effect.

## Introduction

Numerous studies have demonstrated that stories carried by the local and national press can influence the public’s perceptions, attitudes and behaviours [1]–[5]n relation to a health threat. Some evidence also suggests that health-related news studies can have a direct effect on people’s well-being. To a large extent, this relates to the impact reporting can have on someone’s psychological state: articles can be reassuring or provoke anxiety. However, media coverage about a health risk can also contribute to physical symptoms, by causing some people to monitor for, find, and fixate on pre-existing symptoms that might otherwise have gone undetected. For example, two experiments have shown that participants who have read alarming reports about the potential health effects of chemicals are more likely to experience physical symptoms when subsequently exposed to an innocuous odour [6] and that watching a television documentary which highlights the possible health effects of wifi can increase the chances of someone experiencing symptoms following exposure to a sham wifi signal and of subsequently believing that they may be particularly sensitive to wifi [7].

One implication of these findings is that for some conditions, media reporting might be one of the main causes of ill health among the population [8]. A condition where this may be true is idiopathic environmental intolerance attributed to electromagnetic fields (IEI-EMF). This condition is typified by subjective symptoms that occur when an individual perceives themselves to have been exposed to the electromagnetic fields produced by, for example, mobile phones, computers or overhead powerlines. It is more commonly called ‘electrosensitivity’ or ‘electromagnetic hypersensitivity’ in the lay press, however the term IEI-EMF is preferred as being aetiologically more neutral [9]. The condition can result in severe consequences for those affected and in extreme cases people have been known to retreat almost entirely from modern society in order to avoid the electromagnetic fields that seem to trigger their symptoms [10]. Yet despite the conviction of patients that electromagnetic fields are the cause of their ill-health, dozens of well-designed double-blind experiments have failed to produce any convincing evidence that exposure to electromagnetic fields triggers symptoms or physiological effects in people with IEI-EMF [11]–[14]. Most expert groups, including the UK’s Advisory Group for Non-Ionising Radiation [15] and the World Health Organization [16], agree that electromagnetic fields are probably not the cause of the condition. Instead, studies have shown that believing that one has been exposed to electromagnetic fields is sufficient to trigger the symptoms associated with IEI-EMF, regardless of whether or not exposure has actually occurred, suggesting an important role for psychological processes culminating in a ‘nocebo effect’ [17]. Evidence concerning the most appropriate treatments for the condition corresponds with this: while reducing electromagnetic fields provides no more than a placebo effect for sufferers, cognitive behaviour therapy may provide more in the way of long-term benefit, although better studies of the efficacy of psychologically-oriented treatments are still needed [18].

Evidence contradicting an electromagnetic cause for IEI-EMF is not new. In 1997 a report for the European Commission concluded that “electromagnetic hypersensitive people do not react in…provocation studies” [19], while 2005 saw the publication of two influential, independent systematic reviews that “could find no robust evidence to support the existence of a biophysical hypersensitivity to [electromagnetic fields]” [12]; [14]. Nonetheless, rates of IEI-EMF have continued to increase in Britain, from being almost non-existent in 1997 [19] to affecting up to 4% of the population in 2007 [20].

### Study Aims

In this paper we assess whether newspaper reporting in Britain inaccurately portrays IEI-EMF as primarily triggered by electromagnetic fields. We also assess whether newspaper reports generally endorse treatments based on reducing exposure to electromagnetic fields rather than those that address psychological factors, whether the sources that are quoted by an article can influence the way in which the IEI-EMF is portrayed and whether reporting about the condition differs between broadsheet, tabloid and local or regional newspapers.

## Method

### Search for Newspaper Articles

We searched the Nexis® database for British newspaper articles relating to IEI-EMF. This database, to which our institution subscribes, contains a comprehensive, searchable, archive of reports from all major UK national and regional newspapers, including links to the full text of these reports. To ensure our search would identify most relevant articles, we used the following search term: (electrosensitiv! or (allerg! w/5 electricity) or (electr! w/5 (sensitivity or hypersensitivity))), where “!” indicated that the root term as well as that term followed by any number of additional letters would be located and where “w/5” meant that the two relevant words needed to be within five words of each other in order to be located by the search. We also tested two additional searches. First, we tested a search using (‘allerg! electr!’ w/5) which dramatically decreased the specificity of the search and was therefore abandoned. Second, because we were concerned that articles which use the formal scientific name for the condition might have been missed by our search and might take a qualitatively different perspective from those which use colloquial terms, we also searched the websites of *The Guardian*, *The Telegraph*, *The Times*, *The Daily Mail* and *The Mirror* using variations on the phrase ‘idiopathic environmental intolerance attributed to electromagnetic fields:’ this found no additional articles.

### Inclusion Criteria and Selection of Articles

To be included in our analysis, articles had to discuss IEI-EMF and be published in British newspapers between 1 January 2006 to 31 December 2011. We selected 1 January 2006 as our cut-off to ensure that all articles had been published after the release of two systematic reviews assessing all good-quality experimental evidence relating to an electromagnetic cause for IEI-EMF (12;14). We excluded ‘letters to the editor.’

The two authors independently assessed the first 25 articles identified from the search against these criteria and produced identical assessments for all of them. The second author then assessed the remaining articles against the inclusion criteria. For pragmatic reasons, we elected not to analyse all of the articles which met the inclusion criteria. Instead, after producing a list of all articles which met our criteria, a random numbers generator was used to select a random sample of 60 articles to analyse in depth.

### Data Coding

For each article, we assessed what the article as a whole suggested was the cause of IEI-EMF, what treatments were discussed and what sources of information were mentioned.

For causes, we coded articles as ‘cause not mentioned,’ ‘only non-electromagnetic causes discussed,’ ‘only electromagnetic causes discussed,’ or ‘both electromagnetic and non-electromagnetic causes discussed.’ For this last category, a further decision was made as to whether the main emphasis was on electromagnetic or non-electromagnetic causes or whether no main emphasis could be found. These decisions were subjective. Nonetheless, they were usually relatively straightforward to make. For instance, an article entitled, “I quit job over Wi-Fi sickness” explained that, “Ryan, 35, has a condition called electrosensitivity (ES) that means electromagnetic fields (EMF) emitted by everyday gadgets make him sick” although “doctors attribute the symptoms to flu or viruses, or claim they are psychosomatic.” Although this article was classified as presenting both electromagnetic and non-electromagnetic explanations, six out of its seven paragraphs contained descriptions of Ryan’s “misery” which “coincided with the arrival of a new Wi-Fi system” and we therefore categorised this as placing most emphasis on an electromagnetic cause. In contrast, although another article stated that “there is growing evidence that a very small number of people are in fact sensitive to [electromagnetic] radiation,” this statement was more than balanced by repeated statements such as “there is no evidence that Wi-Fi is harmful to our health” and we categorised this article as placing most emphasis on a non-electromagnetic cause.

For treatments, we noted whether articles discussed psychological treatments (e.g. counselling or cognitive behaviour therapy), strategies intended to reduce electromagnetic exposure (e.g. changing jobs or moving house to avoid electromagnetic fields, or using shielding material or devices to reduce exposure), or complementary and alternative interventions (e.g. homeopathy or detoxification treatments). Where treatments were discussed by people with IEI-EMF, we only included them if they had already used that treatment. For example, if someone mentioned that they might have to move house if their condition worsened, we did not include that in the analysis.

For sources of information, we noted what sources were explicitly quoted or referred to in an article. Sources of information were coded as ‘university scientist or researcher,’ ‘official body’ (for example the UK’s Health Protection Agency or Department of Health), ‘lobbying group or charity,’ ‘complementary or alternative therapist,’ ‘another news report,’ ‘politician,’ ‘medical practitioner,’ or ‘person with IEI-EMF.’

The type of newspaper (broadsheet, tabloid or local and regional) was also noted for each article.

### Statistical Analyses

We used binary logistic regressions to calculate the univariate odds ratios for the associations between the cause of, or treatment for, IEI-EMF, and the type of source of information used in an article or the type of newspaper it was published in. For those analyses which included the ‘cause’ variable, we excluded articles which did not mention any cause at all.

## Results

**Figure 1:**
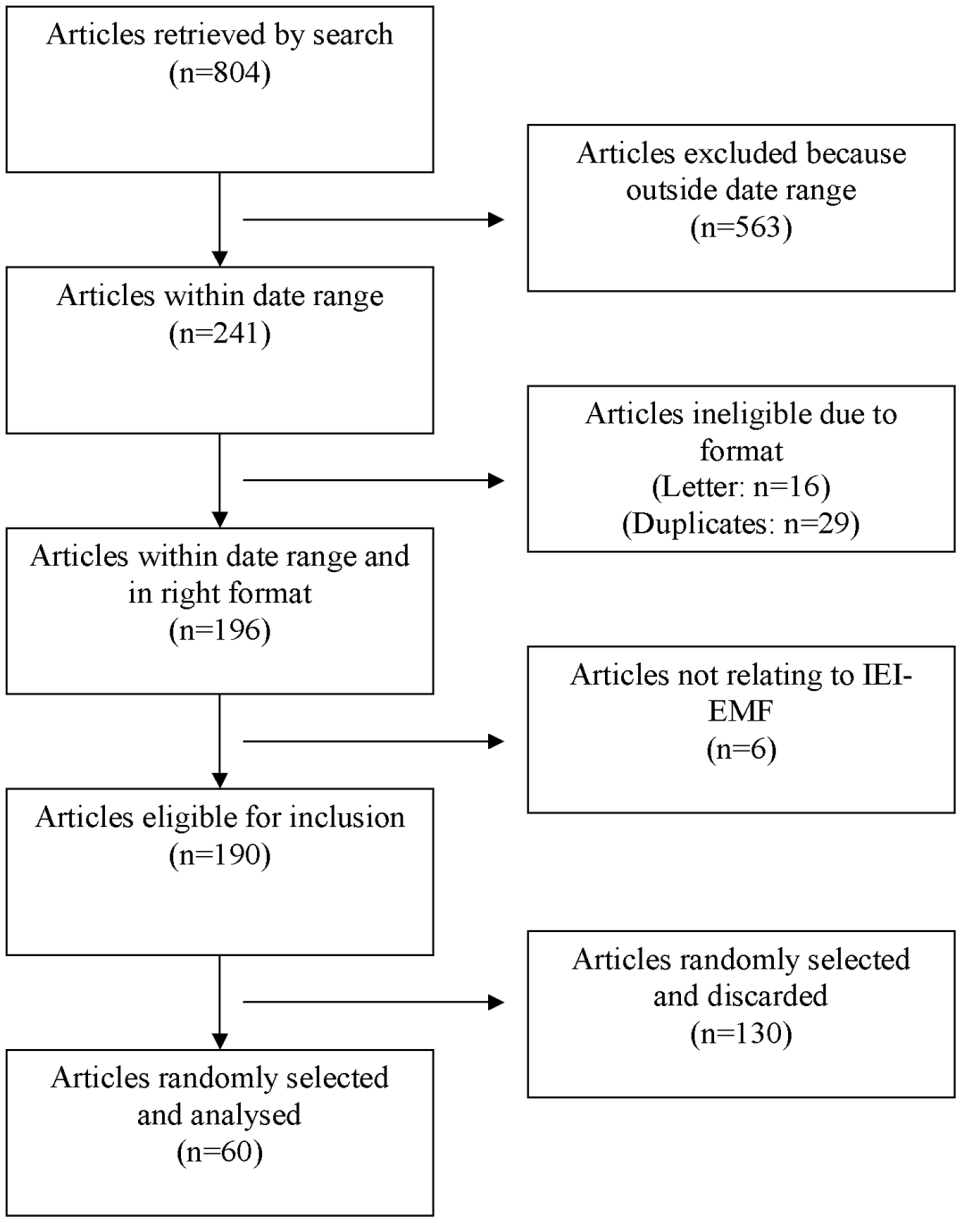
Flow diagram for the application of inclusion criteria to the articles identified in our search.

**Figure 2:**
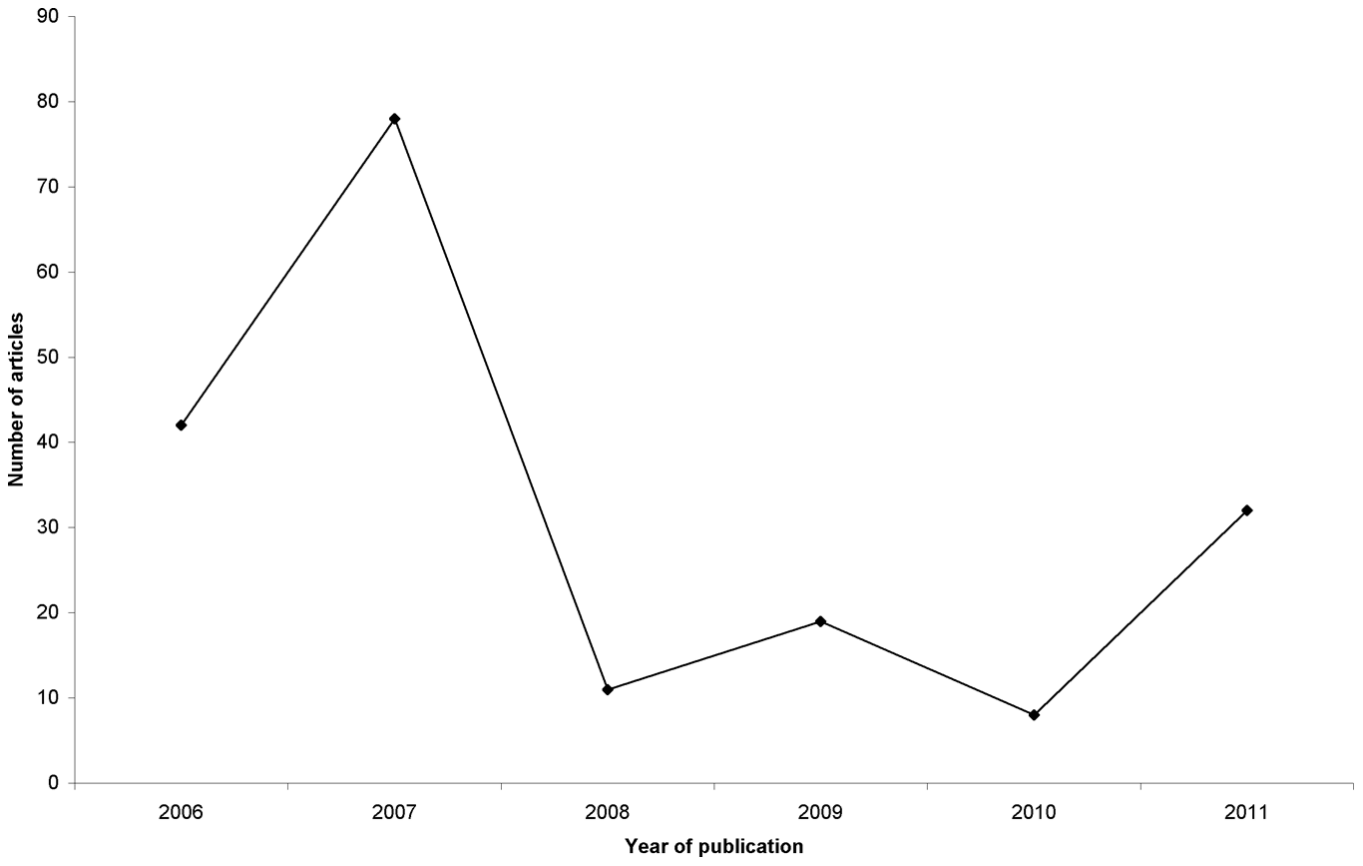
Year of publication for all 190 articles about idiopathic environmental intolerance attributed to electromagnetic fields.

The search retrieved 804 articles published since 1984. 563 were excluded because they were outside our date range. Of the remainder, 16 were then excluded because they were letters and 29 were excluded because they were duplicates of articles already identified in the search. Finally, six articles were excluded because they did not relate to IEI-EMF. 190 articles met our criteria in full. The 60 articles that we randomly selected from this list therefore represented 31.6% of all articles published during the period. Figure 1 presents a flow diagram illustrating the process of the search and the application of the exclusion criteria. Figure 2 shows the dates of publication for all 190 papers that met our inclusion criteria in full.

Nine of the articles analysed in full were published by broadsheet newspapers, 16 by tabloids and 35 by local or region newspapers.

### Causes of IEI-EMF

Of the 60 articles, 4 (6.7%) did not discuss the cause of IEI-EMF, 10 (16.7%) only presented IEI-EMF as having a non-electromagnetic cause, 32 (53.3%) only presented IEI-EMF as having an electromagnetic cause and 14 (23.3%) mentioned both electromagnetic and non-electromagnetic causes. Of the 14 which considered both possible causes, 3 (21.4%) placed most emphasis on non-electromagnetic causes and 11 (78.6%) on electromagnetic causes. Overall, then, 43 articles (71.7%) presented a mainly electromagnetic cause, compared to 13 (21.7%) which presented mainly non-electromagnetic causes and 4 (6.7%) which did not discuss the cause at all.

Typical quotes from articles which presented non-electromagnetic causes included, “mobile phone signals are not responsible for the symptoms that some people described;” “carefully designed experiments showed that symptoms are likely to be psychosomatic” and “there is no scientific basis to link [electromagnetic hypersensitivity syndrome] symptoms to [electromagnetic] exposure.” Articles that only presented IEI-EMF as an electromagnetic condition typically contained quotes such as “[she] says radiation from electrical equipment like mobile phones and microwave ovens can make her seriously ill,” “national charity Electrosensitivity UK is independently assessing risks associated with electromagnetic radiation [which] has serious cumulative effects on a few [people]” and “Wi-Fi makes me feel like I have a clamp at the back of my head which is squeezing the life out of me.”

### Treatments for IEI-EMF

29 articles (48.3%) did not mention any potential treatment for IEI-EMF, while 24 (40.0%) mentioned interventions intended to reduce electromagnetic exposure and 12 (20.0%) mentioned complementary or alternative therapies. None mentioned psychological treatments.

Many of the articles which mentioned interventions intended to reduce electromagnetic exposures (including protective screening material or devices) advocated their remedial effects. For example, one lady was described whose “silver-coloured balaclava and matching gloves” had allowed her “to re-enter the world after an 18-year exile. The suit has changed her life,” while another article described a person who had bought “special rolls of foil wallpaper and a fabric called Swiss bobbinet” which “promised to ‘shield’ her from any emissions from phone masts or wireless broadband systems… Within a few weeks of the wallpaper going up and the windows being hung with netting, she began to feel better.” Lifestyle changes were also identified in this category and were, again, presented as largely effective because, in the words of one interviewee, “the only cure is avoiding EMF.” A typical story described one person with IEI-EMF who “went away for the Easter break to stay with friends in the depths of remote countryside” where she “felt great.” This experience prompted her to remove her Wi-Fi at home and to “bin the dect [digital] phones.” Another typical story described a sufferer who “rented a house away from the mast” which had caused her “blinding headaches.” On doing this, she “switched off the power and found some relief.”

### Sources of Information

People with IEI-EMF were the most frequently used sources of information (mentioned in 30 articles, 50%), followed by a charity or lobby group (29, 48.3%), scientists (22, 36.7%), official organizations (19, 31.7%), medical practitioners (10, 16.7%), politicians (6, 10.0%), other media sources (5, 8.3%) and complementary or alternative therapists (3, 5.0%).

Table 1 shows the associations between the source of information used in an article and whether the article mainly presented the cause of IEI-EMF as being due to electromagnetic fields, whether they discussed treatment strategies based on reducing exposure to electromagnetic fields and whether they discussed complementary and alternative therapies. Articles which used a scientist as a source of information were significantly less likely to present electromagnetic fields as the cause (odds ratio: 0.1, 95% confidence interval 0.03 to 05). Those which used a person with IEI-EMF as a source were substantially more likely to suggest electromagnetic fields as the cause (10.3; 2.0 to 52.5) and to discuss strategies intended to reduce exposure to electromagnetic fields (38.5; 7.5 to 199.9) and complementary and alternative therapies (16.8; 2.0 to 140.9) as interventions. No other associations were significant.

**Table 1:** Association between source of information used in a newspaper report and whether the report supported an electromagnetic cause for idiopathic environmental intolerance attributed to electromagnetic fields (IEI-EMF) or suggested that strategies to reduce exposure to electromagnetic fields (EMF) or complementary and alternative therapies might be helpful interventions.

Source of information	Number (%) of articles out of 60 using that source	Association with attributingcause of IEI-EMF to EMF(Odds ratio (95%confidence interval))	Association with presenting strategies to reduce EMF exposure (Odds ratio (95% confidence interval))	Association with presenting complementary and alternative therapies (Odds ratio (95% confidence interval))
Scientist	22 (36.7%)	0.1 (0.03 to 0.5)	0.6 (0.2 to 1.7)	0.8 (0.2 to 3.2)
Official body	19 (31.7%)	1.6 (0.4 to 6.8)	0.8 (0.3 to 2.5)	0.4 (0.1 to 1.9)
Lobby group or charity	29 (48.3%)	0.7 (0.2 to 2.6)	0.5 (0.2 to 1.4)	0.3 (0.07 to 1.2)
Complementary or alternative therapist	3 (5.0%)	0.6 (0.06 to 7.0)	3.2 (0.3 to 37.2)	2.1 (0.2 to 25.2)
Another news report	5 (8.3%)	0.2 (0.02 to 1.1)	2.4 (0.4 to 15.8)	3.0 (0.4 to 20.4)
Politician	6 (10.0%)	1.2 (0.1 to 12.1)	0.3 (0.03 to 2.5)	0.8 (0.08 to 7.4)
Medical practitioner	10 (16.7%)	Not calculated^a^	2.7 (0.7 to 10.7)	3.5 (0.8 to 15.3)
Person with IEI-EMF	30 (50.0%)	10.3 (2.0 to 52.5)	38.5 (7.5 to 199.9)	16.8 (2.0 to 140.9)

a :Odds ratio not calculated as all articles using a medical practitioner as a source attributed the cause of IEI-EMF to EMF.

No associations were found between type of newspaper and any of the three outcome variables (all p-values >0.17).

## Discussion

In Britain, newspaper reporting about IEI-EMF is substantially out of step with the current scientific evidence about the condition. In 2005, two systematic reviews assessing the evidence from dozens of well-designed double-blind experimental studies concluded that no convincing evidence existed to show that IEI-EMF was related to the presence of electromagnetic fields [12]; [14]. Since then, several additional experiments have added strength to this conclusion [11]; [13]. Yet around three quarters of newspaper reports over same period have conveyed the opposite message to the public: that IEI-EMF is probably caused by exposure to man-made electromagnetic fields. Similarly, while limited evidence currently exists on possible treatments for the condition, several experiments have demonstrated that interventions intended to reduce exposure to electromagnetic fields provide no more than a placebo effect. Yet 40% of the newspaper reports that we analysed suggested that beneficial effects could be achieved through interventions intended to reduce electromagnetic fields, while 20% described beneficial effects from complementary or alternative treatments or from screening material.

This mismatch between scientific evidence and media reporting is disappointing, though as expected. Previous analysis of reporting on other forms of controversial medical issues, including chronic fatigue syndrome/ME have produced similar findings [21]. However, we believe that this poor reporting is particularly important in the context of IEI-EMF, for two reasons. On one level, it represents a missed opportunity to inform members of the public about how scientific research can used to test the validity of strongly-held beliefs and how physical symptoms can sometimes have an underlying psychological component. On another level it is possible that by endorsing an electromagnetic cause for the symptoms described by people with IEI-EMF, the media have directly contributed to the growing prevalence of the condition. This effect could occur through two mechanisms. First, people with pre-existing symptoms, which may or may not have some other medical explanation, might self-diagnose themselves as having ‘electrosensitivity’ on the basis of media reports. Clinical trials involving people with IEI-EMF have found that between 14% and 33% of patients who report sensitivity to electromagnetic fields also have some other, more well-established psychiatric or organic illness which might account for their symptoms [17]. Second, by encouraging people to monitor themselves for possible symptoms after exposure to electromagnetic fields, inaccurate media reporting may increase the chances of some people detecting symptoms, focusing on them, attributing them to the exposure and then speculating whether they themselves might be sensitive to electromagnetic fields [7]. A vicious circle of increased concern about electromagnetic fields and increased expectation, and detection, of symptoms following exposure may then ensue.

While experimental evidence has demonstrated that this process is theoretically possible, cross-cultural evidence provides some indication of its importance. In particular, while some countries (including Britain) appear to have increasing rates of IEI-EMF, it is striking that colleagues in other countries, including Iran and India are relatively unaware of people with the condition, something which they attribute to the lack of attention given to the possible health effects of electromagnetic fields by the media in their countries [22], [23]. The importance of changes in media reporting over time in changing how patients describe medically unexplained symptoms has also long been recognized by specialists within specific countries [24].

The poor nature of the reporting that we identified was not restricted to any one sector of the British press: broadsheets, tabloids and regional or local newspapers were equally likely to present an electromagnetic cause for IEI-EMF and to describe disproven or untested interventions. Unsurprisingly, when a person with IEI-EMF was used as a source of information for an article, these issues were more prevalent. Encouragingly, however, where an article included a scientist or university researcher, the description of the condition was more likely to be in accordance with the best currently available evidence. In part, this may have been due to a qualitative difference between articles that included people with IEI-EMF and those that interviewed scientists. While the former were more likely to describe the compelling ‘human interest’ stories of people who have been severally affected by the condition, the latter seemed more likely to be driven by university press releases discussing the results of a new study in this area. Indeed, several of the news reports that we included focused on the findings of one double-blind experiment in the UK which demonstrated that IEI-EMF was not associated with exposure to mobile phone mast signals [25]. The publication of this experiment was accompanied by a peak in newspaper reporting in the year in which it was published (2007: see Figure 2). Nonetheless, our results should encourage scientists working in controversial areas such as this to stay actively involved with the media.

### Methodological Limitations

Three main limitations should be considered for this study. First, the relatively small number of articles that we assessed may have restricted our ability to identify small associations between the variables. Replication using a larger sample may be worthwhile.

Second, there exists a possibility that our results were skewed by the inclusion of several articles that were all the result of a press release and briefing relating to a single influential study. Had this event not occurred, our results would have suggested even more strongly that press reporting in the UK does not reflect current scientific evidence.

Finally, for pragmatic reasons we only analyzed newspaper reporting. It is possible that had we included television, radio or internet reporting, a different pattern of results would have emerged. We believe this is unlikely, however, and are aware of no compelling evidence that reporting in other forms of media is qualitatively better on this issue.

### Conclusions

Our analysis of a representative sample of newspaper reports about IEI-EMF in Britain suggests that substantial room for improvement exists in how journalists convey the science in this area. We would urge scientists to remain engaged with journalists in order to help improve the quality of reporting.
